# β-Aminopropionitrile monofumarate induces thoracic aortic dissection in C57BL/6 mice

**DOI:** 10.1038/srep28149

**Published:** 2016-06-22

**Authors:** Weihong Ren, Yan Liu, Xuerui Wang, Lixin Jia, Chunmei Piao, Feng Lan, Jie Du

**Affiliations:** 1 Beijing Anzhen Hospital, Affiliated to Capital Medical University; Collaborative Innovation Center for Cardiovascular Disorders; The Key Laboratory of Remodeling-Related Cardiovascular Diseases, Ministry of Education; Beijing Institute of Heart Lung and Blood Vessel Diseases, Beijing 100029, China

## Abstract

Thoracic aortic dissection (TAD) is a catastrophic disease with high mortality and morbidity, characterized by fragmentation of elastin and loss of smooth muscle cells. However, the underlying pathological mechanisms of this disease remain elusive because there are no appropriate animal models, limiting discovery of effective therapeutic strategies. We treated mice on C57BL/6 and FVB genetic backgrounds with β-aminopropionitrile monofumarate (BAPN), an irreversible inhibitor of lysyl oxidase, for 4 wk, followed by angiotensin II (Ang II) infusion for 24 h. We found that the BAPN plus Ang II treatment induced formation of aortic dissections in 100% of mice on both genetic backgrounds. BAPN without Ang II caused dissections in few FVB mice, but caused 87% of C57BL/6 mice to develop TAD, with 37% dying from rupture of the aortic dissection. Moreover, a lower dose of BAPN induced TAD formation and rupture earlier with fewer effects on body weight. Therefore, we have generated a reliable and convenient TAD model in C57BL/6 mice for studying the pathological process and exploring therapeutic targets of TAD.

Thoracic aortic dissection (TAD) ranks among the most lethal vascular diseases, occurring at a rate of 3 cases per 100,000 individuals per year. Blood enters into the media space of thoracic aorta, leading to separation of the layers within the aortic wall, which characterizes TAD. Its key pathologic feature is medial degeneration, a process characterized by smooth muscle cell depletion and extracellular matrix degradation^1^. However, the molecular mechanisms of medial degeneration remain elusive because there is no stable, convenient model of TAD^2^.

Most of the existing mouse models reported for TAD were caused by genetic mutation, such as in fibrillin-1, TGBβR, SMAD3 or ACTA2^3^,^4^. Besides these genetic models, surgery and drug treatment have also been used to induce TAD. For example, either long-term angiotensin II (Ang II) infusion using apoE−/− mice or continuous perfusion of elastase, resulted in abdominal aortic aneurysm (AAA) or abdominal aortic dissection (AAD)^5^,^6^,^7^. However, the rate of TAD induction is very low in these models. Thus, there is no appropriate TAD mouse model for studying the mechanism of TAD.

Recently, it was reported that a 4-wk administration of β-aminopropionitrile monofumarate (BAPN), an inhibitor of lysyl oxidase (LOX), combined with a 24-h infusion of Ang II, induced aortic dissection (AD) rupture in wildtype FVB mice, while BAPN administration alone caused dissection in about 10% of these mice^8^. In another study, LOX-null C57BL/6 mice developed aortic rupture spontaneously^9^,^10^ and in our recent report, BAPN administration alone induced TAD in wildtype C57BL/6 mice^4^. In this study, we systemically evaluated how BAPN can induce TAD in mice and examined its pathologic characteristics.

## Results

### Basal characterization of mice with BAPN treatment, with or without Ang II

**Figure 1:**
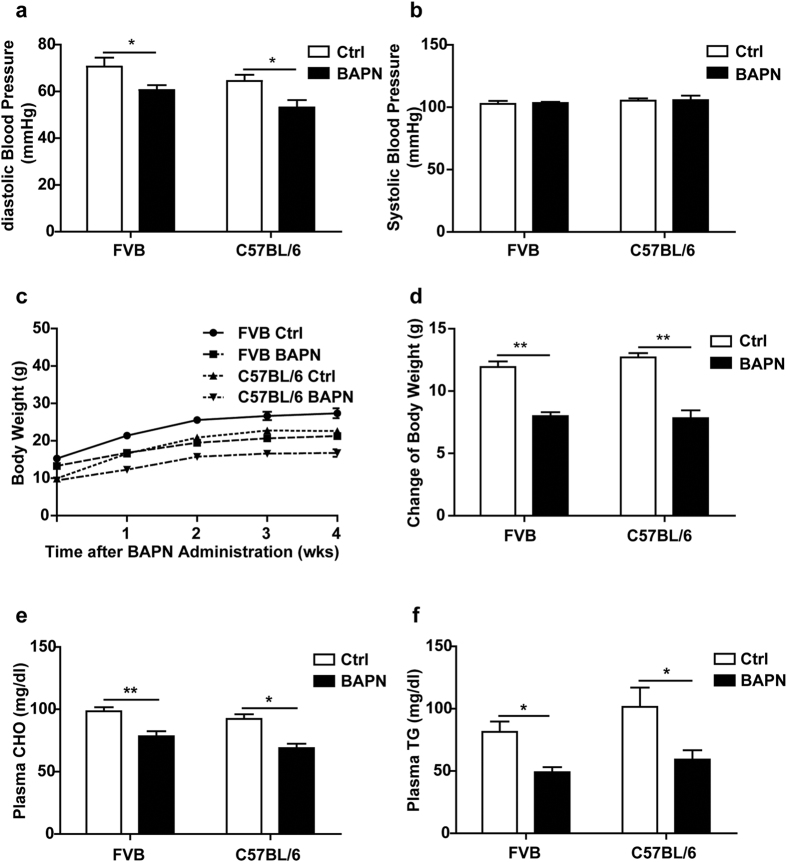
Basal parameters of BAPN treatment. C57BL/6 and FVB mice were administered vehicle or BAPN for 4 wk. (**a,b**) Diastolic and systolic blood pressures (n = 6 per group); *P < 0.05. (**c,d**) Body weights with bar graph showing changes during the study (n = 6 per group); **P < 0.01. (**e,f**) Levels of plasma cholesterol (CHO) (n = 11 for FVB Ctrl, n = 16 for FVB BAPN, n = 6 for C57BL/6 Ctrl, n = 15 for C57BL/6 BAPN) and triglyceride (TG) (n = 12 for FVB Ctrl, n = 6 for FVB BAPN, n = 7 for C57BL/6 Ctrl, n = 15 for C57BL/6 BAPN); *P < 0.05, **P < 0.01.

It was reported that treatment with the LOX inhibitor BAPN plus Ang II induced TAD in FVB mice^8^. We recently found that, on a C57BL/6 background, the same dose of BAPN caused sudden death in approximately 56% of mice, prior to Ang II administration, and that this was caused by thoracic aortic ruptures^4^. We thus compared the effects of BAPN in mice with the two genetic backgrounds. Male mice (3 wk old) on FVB or C57BL/6 backgrounds were administered BAPN in drinking water at a dose of 1 g per kg body weight for 4 wk. BAPN treatment reduced diastolic (Fig. 1a) with no effect on systolic (Fig. 1b) blood pressure, indicating increased aortic stiffness. BAPN treatment also attenuated body weight gains (Fig. 1c,d) and significantly decreased plasma triglyceride and cholesterol levels (Fig. 1e,f) in both FVB and C57BL/6 mice.

### Induction of TAD with BAPN treatment

**Figure 2:**
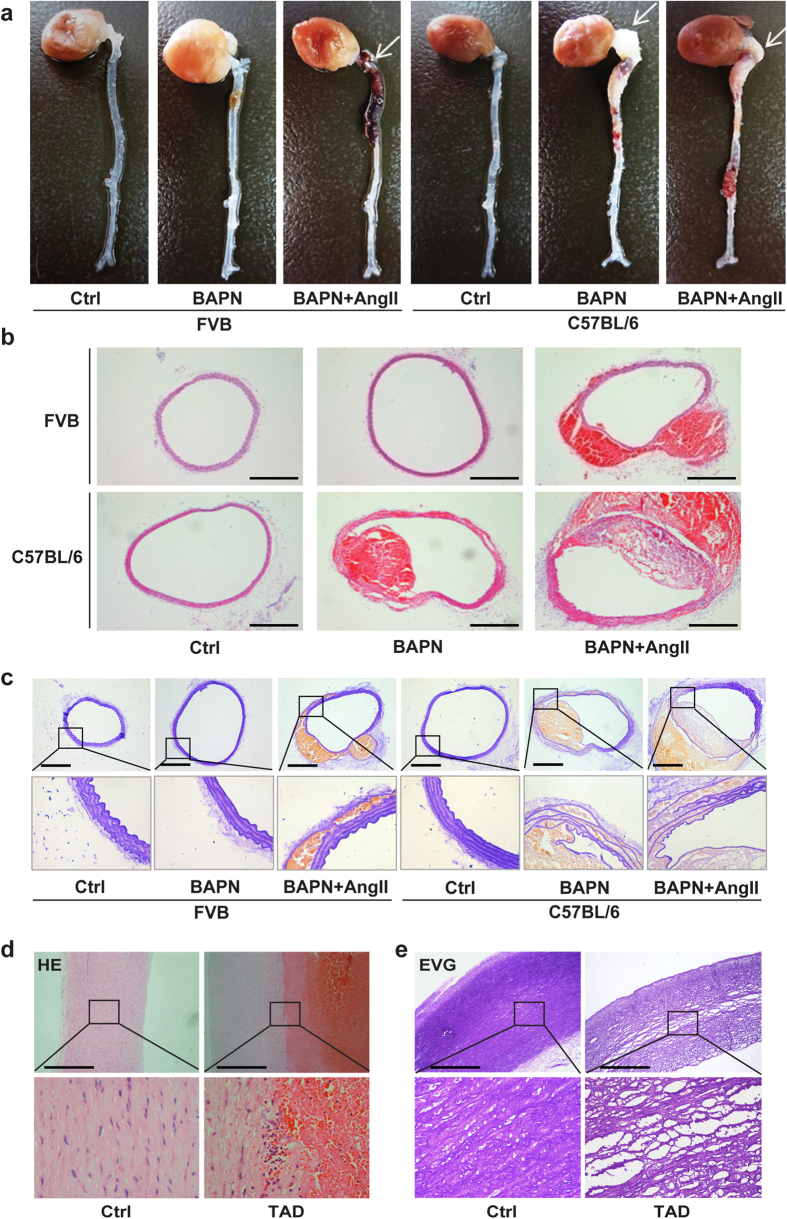
BAPN treatment, without Ang II infusion, induced TAD in C57BL/6 mice, reproducing major features of human TAD. (**a**) Representative images showing macroscopic features of isolated mouse aorta after vehicle or BAPN treatment for 4 wk; white arrow indicates location of TAD. (**b**) Representative haematoxylin and eosin (HE) staining showing blood clot inside torn aortic wall after BAPN administration. (**c**) Representative Elastica Van Gieson (EVG) staining presenting elastic fragmentation and loss of smooth muscle cells after BAPN administration. Representative HE (**d**) and EVG (**e**) staining in human samples. Scale bar: 50 μm.

We next examined the effects of BAPN treatment on the incidence of TAD. To determine whether Ang II was also required for TAD development, the mice were sacrificed for autopsy after 4 wk of BAPN treatment, with or without 24 h of Ang II infusion. Consistent with the previous report^8^, administration of BAPN plus Ang II induced TAD in all mice, while approximately 75% of FVB mice treated with BAPN alone did not develop TAD (Fig. 2a and Table 1). However, the incidence of TAD in C57BL/6 mice treated with only BAPN reached 87% (Fig. 2a and Table 1) and 45% of mice in this group died of aortic rupture. TAD was also observed in all C57BL/6 mice treated with BAPN plus Ang II, with 50% of these having aortic rupture within 24 h of Ang II infusion. Aortas of C57BL/6 mice given BAPN, with or without Ang II infusion, were enlarged from the root to the thoracic segment and, in some cases, the abdominal segment was also involved. Hematomas were observed in the lesions, indicating thrombosis (Fig. 2a). Haematoxylin and eosin staining showed tearing of the aortic wall and thrombi in the false lumens (Fig. 2b). Massive fragmentation and depletion of elastic fibres and smooth muscle cell loss were confirmed by Elastica van Gieson (EVG) staining (Fig. 2c). All these pathological changes resembled those observed histopathologically in humans (Fig. 2d).

### BAPN dose optimization for TAD induction

**Figure 3:**
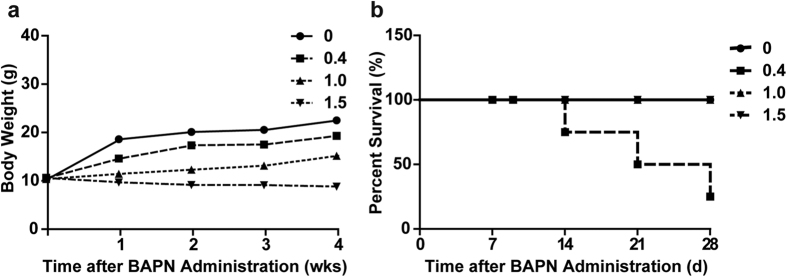
Dose-dependence of BAPN-induced TAD in C57BL/6 mice. C57BL/6 mice were equally segregated into four weight-matched groups and administered diets containing 0, 0.4, 1.0 or 1.5 g BAPN per 100 g diet, respectively, for 4 wk (n = 6 per group). (**a**) Body weights measured weekly. (**b**) Kaplan-Meier survival curves.

To further investigate the causal effects of medial degeneration on TAD formation, we applied different doses of BAPN by feeding 3-wk-old C57BL/6 male mice with diets containing 0, 0.4, 1.0 or 1.5 g BAPN per 100 g mouse chow for 4 wk. Body weights were lower with increased BAPN doses (Fig. 3a). All six mice fed with the 0.4 g BAPN per 100 g diet developed TAD and five died of dissection ruptures by 2 to 4 wk after BAPN administration. Of six mice fed with the 1.0 g BAPN diet, two had TAD at the end of the treatment, but no ruptures occurred. Most surprisingly, no TAD formation was observed in mice fed with the 1.5 g BAPN diet (Fig. 3b).

### Molecular phenotypic features of BAPN-induced TAD

**Figure 4:**
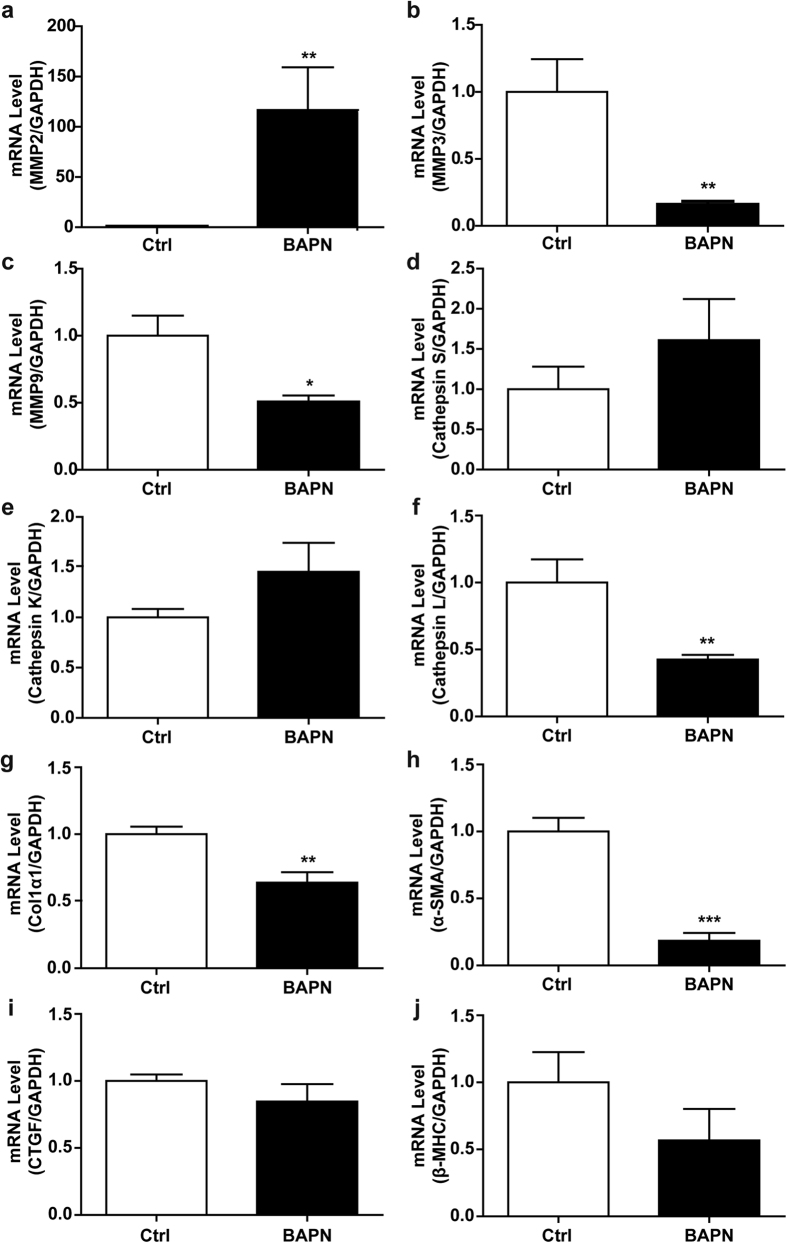
Phenotype-related gene expression in aortic media of BAPN-treated mice. C57BL/6 mice were administered vehicle or BAPN for 4 wk. (**a–c**) qPCR analysis of MMP2/3/9 mRNA levels; n = 8, *P < 0.05, **P < 0.01. (**d–f**) qPCR analysis of Cathepsin S/K/L mRNA levels (n = 8 per group); *P < 0.05, **P < 0.01. (**g–j**) qPCR analysis of COL1α1, α-SMA, CTGF and β-MHC mRNA levels (n = 8 per group); *P < 0.05, **P < 0.01, ***P < 0.0001.

Because BAPN-induced TAD exhibited typical histological features of the human disease, we next examined whether expression of TAD-related genes were also changed in the media of aortas. A panel of genes known to be dysregulated during medial degradation in TAD formation were selected for analysis. These were matrix metalloproteinases (MMPs, MMP2/3/9)^5^,^8^,^11^,^12^ and cathepsins (cathepsin S/K/L)^13^ (that degrade extracellular matrix), collagen I α1 (COL1α1) and connective tissue growth factor (CTGF) (target genes indicating activation of the TGF-β signalling pathway in LDS)^14^, α-smooth muscle actin (α-SMA) and β-myosin heavy chain (β-MHC) (associated with familial thoracic aortic aneurysm and dissection syndrome)^15^. Expression of these genes was compared in control and BAPN-treated C57BL/6 mice. In the BAPN-treated group, compared with the control, MMP2 was significantly upregulated (Fig. 4a), while MMP3 and MMP9 were downregulated (Fig. 4b,c). Cathepsin S and cathepsin K levels were no different in the two groups (Fig. 4d,e), while cathepsin L was significantly decreased in the BAPN group (Fig. 4f). Both COL1α1 and α-SMA expression were dramatically decreased with BAPN treatment (Fig. 4g,h), while CTGF and β-MHC levels were not changed (Fig. 4i,j). These results revealed that BAPN-induced TAD was associated with typical ECM degradation, possibly via MMP2, and loss of SMC leading to decreased α-SMA, effects consistent with previous observations in humans and mouse models.

## Discussion

Thoracic aortic dissection, with or without rupture, represents a structural and functional failure of the aortic wall and can occur when integrity of the thoracic aortic wall is impaired^15^. In our study, we found that at the proper dose (0.4 g BAPN per 100 g diet), BAPN disrupted the aortic medial layer without substantially affecting metabolism and body growth, effectively inducing TAD in C57BL/6 mice, a widely used strain. We showed that TAD in C57BL/6 mice resembled clinically observed TAD, with a high incidence of rupture and mortality.

Clinically, TAD, without exception, exhibits medial degeneration, which results from aortic wall stressors such as hypertension, genetics and inflammatory conditions^15^. Medial degeneration is, therefore, essential for rupture of the intima or haemorrhage within the media. Thus, acute blood pressure elevation, such as hypertension induced by Ang II perfusion, is likely to trigger, but not serve as a pathological mediator of, TAD and rupture. Moreover, in a previous report, norepinephrine infusion in BAPN-treated mice failed to induce TAD, despite elevating blood pressure similarly to Ang II infusion. This suggested that the effects of Ang II on triggering TAD onset were not caused by a blood pressure change alone^8^. To further support this, we found that 87% mice treated only with BAPN developed thoracic aortic dissection and 37% had spontaneous rupture without elevation of systolic blood pressure. As expected, thoracic aortic dissection and rupture were observed in 100% of mice receiving both BAPN and Ang II on both genetic backgrounds, supporting a triggering effect of Ang II after aortic structural disruption by BAPN. Loss of elasticity resulted in decreased diastolic blood pressure^16^, explaining our observation of reduced diastolic blood pressure in mice of both genetic backgrounds given BAPN alone, as compared with controls. Interestingly, spontaneous rupture occurred with mice given the lower doses, but not the higher doses, of BAPN. These mice also showed limited weight increases over time, suggesting that a sufficient hemodynamic load was required for the progression of TAD. Further investigation will be needed to understand the mechanisms involved in this mouse model for BAPN-induced TAD.

## Methods

### Patient specimens and ethics statement

Dissection samples were collected from TAD patients who had undergone repair surgery in Beijing Anzhen Hospital. None of the patients had a known genetic syndrome related to aortic diseases, such as Marfan’s, Turner’s, Loeys–Dietz or Ehlers–Danlos syndromes. Control aortas were trimmings that had been discarded during heart transplantation surgeries. Informed consent was obtained for use of these specimens. Use of human tissue was approved by the Medical Ethical Committee of Capital Medical University, Beijing, and complied with the principles outlined in the Declaration of Helsinki^4^.

### Animal model and ethics statement

Wildtype C57BL/6 and FVB mice were obtained from HFK Bioscience Company (Beijing, China). All studies were reviewed and approved by The Institutional Animal Care and Use Committee of Capital Medical University, Beijing, China. The investigation conformed to the Guide for the Care and Use of Laboratory Animals, published by the US National Institutes of Health (NIH). Three-week-old male mice were fed a normal diet and administered freshly prepared BAPN (Sigma-Aldrich, St. Louis, MO, USA) solution dissolved in the drinking water (1 g/kg/d) for 4 wk, as described previously^8^. Blood pressure was measured before and after BAPN administration for 4 wk, using the tail-cuff method. Interventions lasted 4 wk and body weights were measured weekly. As previously reported, at 7 wk old, osmotic mini pumps (Alzet, Cupertino, CA, USA) administering 1 μg/kg per min Ang II (Sigma-Aldrich, St. Louis, MO, USA) were implanted subcutaneously and mice were euthanized 24 h after implantation^17^. All mice died before expected end time of the experiment were autopsied immediately, and Blood clots were found in the thoracic cavities of these mice. Mice surviving at the end of the experiment were sacrificed by an overdose of sodium pentobarbital and their blood and tissue samples were collected for further analyses.

### Histopathological analysis

Complete gross and histopathological evaluations were performed with samples from control and BAPN-treated mice. After euthanasia, normal and dissected aortas were harvested from the ascending aorta to the iliac artery and were fixed in 10% buffered formalin, as were human tissues. Fixed, paraffin-embedded tissues were cut at 5 μm thickness, stained with haematoxylin and eosin following standard procedures and examined under light microscopy, as previously described^4^.

### Elastin staining

Elastin in the normal and dissected aortas was stained with the Gomrori’s aldehyde-fuchsin staining method, using an elastic fibre staining kit (Maixin Bio, Fuzhou, China) as previously described^4^. Briefly, after deparaffinization and rehydration, sections were incubated for 5 min in Lugol’s iodine solution, washed with PBS and incubated with sodium thiosulfate for 5 min. After washing with PBS and 70% ethanol, sections were incubated with aldehyde-fuchsin for 10 min and acid Orange G for s.

### Plasma lipid measurements

Plasma levels of triglyceride (TG) and cholesterol (CHO) were measured by COD-PAP and GPO-PAP methods, respectively, with automated clinical chemistry analyser kits (Biosino Biotech, Beijing, China).

### Quantitative real-time polymerase chain reaction (RT-PCR)

Total RNA of aortic media without thrombi was isolated by the acid-phenol extraction method in the presence of chaotropic salts (Trizol, Thermo Fisher, Carlsbad, CA, USA) and subsequent isopropanol-ethanol precipitation, as described previously^18^. RNA (2 μg) was reverse-transcribed using the GoScript™ Reverse Transcription System (Thermo Fisher, Carlsbad, CA, USA), according to the manufacturer’s instructions. For real-time quantitative PCR, the iQ5 system (Bio-Rad, Hercules, CA, USA) with SYBR Green I (Takara, Shiga, Japan) was used. Amplification was performed at 95 °C for 5 min, 95 °C for 45 s and 60 °C for 1 min, for 45 cycles. The housekeeping gene glyceraldehyde 3-phosphate dehydrogenase (GAPDH) was used as a control. The primers used are listed in Table 2.

### Statistical analysis

In all experiments, data were collected form more than 6 mice per group to calculate means ± standard deviation (SD). Student’s t-test was used for statistical analysis and *P* < 0.05 was considered statistically significant.

## Additional Information

**How to cite this article**: Ren, W. *et al*. β-Aminopropionitrile monofumarate induces thoracic aortic dissection in C57BL/6 mice. *Sci. Rep.* **6**, 28149; doi: 10.1038/srep28149 (2016).

## Figures and Tables

**Table 1:** Incidence of TAD in mice treated with BAPN, with or without Ang II.

	BAPN		BAPN + AngII	
	FVB	C57BL/6	FVB	C57BL6
AD	25%	87%	100%	100%
TAD	25%	87%	100%	75%
AAD	25%	13%	20%	75%
AD-rupture	0%	37%	20%	50%

AD, aortic dissection; TAD, thoracic aortic dissection; AAD, abdominal aortic dissection.

**Table 2:** Primers for PCR.

Gene name	Oligoes for primer (forward/reverse)
mouse MMP2	5′-CGATGTCGCCCCTAAAACAG-3′ 5′-GCATGGTCTCGATGGTGTTC-3′
mouse MMP3	5′-GTTCTGGGCTATACGAGGGC-3′ 5′-TTCTTCACGGTTGCAGGGAG-3′
mouse MMP9	5′-TGGGCGTTAGGGACAGAAAT-3′ 5′-GAACCATAACGCACAGACCC-3′
mouse Cathepsin S	5′-CATCTTTGGAGTGAGCACCA-3′ 5′-GCATCCAAAACAGCCATCTTA-3′
mouse Cathepsin K	5′-CGAAAAGAGCCTAGCGAACA-3′ 5′-TGGGTAGCAGCAGAAACTTG-3′
mouse Cathepsin L	5′-CAAATAAGAATAAATATTGGCTTGTCA-3′ 5′-TGTAGCCTTCCATACCCCATT-3′
mouse CTGF	5′-TGACCCCTGCGACCCACA-3′ 5′-TACACCGACCCACCGAAGACACAG-3′
mouse β-MHC	5′-ATGTGCCGGACCTTGGAA-3′ 5′-CCTCGGGTTAGCTGAGAGATCA-3′
mouse α-SMA	5′-TCCAGCCATCTTTCATTGGGA-3′ 5′-CCCCTGACAGGACGTTGTTA-3′
mouse COL1α1	5′-GAGCGGAGAGTACTGGATCG-3′ 5′-GTTCGGGCTGATGTACCAGT-3′
mouse GAPDH	5′-ACCCAGAAGACTGTGGATGG-3´ 5′-CACATTGGGGGTAGGAACAC-3′
